# An evaluation of the correlation between haematological biomarkers in cats affected with periodontal disease stages 3 and 4

**DOI:** 10.1002/vro2.70012

**Published:** 2025-05-29

**Authors:** Hossein Ghorbani, Azin Tavakoli, Mohammad Heidarpour, Negin Rahimdoust Mozhdehi, Hossein Kazemi Mehrjerdi

**Affiliations:** ^1^ Department of Clinical Sciences Faculty of Veterinary Medicine Ferdowsi University of Mashhad Mashhad Iran; ^2^ Department of Clinical Sciences Islamic Azad University Garmsar Iran; ^3^ Dental Pet Center Tehran Iran

## Abstract

**Background:**

Periodontal disease is the most common disease of the tooth‐supporting apparatus in dogs and cats. Identifying factors associated with periodontal disease early through minimally invasive, fast and cost‐effective methods, such as a simple blood test, may improve treatment outcomes. The current study investigated the association between haematological biomarkers and periodontal disease in cats.

**Methods:**

Sixty cats were divided into healthy cats (*n* = 30) and cats with periodontal disease (*n* = 30) groups. Age, neutering status and breed were recorded. All cats had no history of infection and other inflammatory or neoplastic diseases. No medication had been administered within the previous 3 months and no cats had undergone recent surgery. According to the American Veterinary Dental College pet periodontal disease staging, all cats in the diseased group were diagnosed with stage 3 or 4 periodontal disease and were candidates for extraction of at least one canine or premolar tooth, given that no incisors were noted for extraction. Blood samples were taken from both groups to obtain a complete blood count. The neutrophil‐to‐lymphocyte, platelet‐to‐lymphocyte and monocyte‐to‐lymphocyte ratios were calculated. The results were statistically analysed between the two groups with an independent sample *T*‐test.

**Results:**

No statistically significant difference was observed between the two groups with regard to the various haematological parameters.

**Conclusion:**

According to the results of this study, no significant differences were observed between haematological biomarkers and periodontal disease stage 3 or 4.

## INTRODUCTION

Inflammation of the periodontium, known as periodontitis, is an active disease state that affects the subgingival region, which is located beneath the free gingival margin in the sulcus or periodontal pocket.^1^, ^2^ Periodontal disease (PD) is a common oral condition affecting dogs and cats.^3^ According to one study, PD affects 80%–85% of dogs and cats older than 2 or 3 years.^4^ In cats, PD is a complex inflammatory condition caused by the interaction between subgingival plaque bacteria and the host immune response, leading to chronic inflammation and tissue destruction.^5^ In humans, a strong indication suggests that the inflamed and ulcerated pocket epithelium provides a direct entry point for oral microorganisms. Brief episodes of bacteraemia are likely to occur multiple times a day. Bacterial endotoxins and microbial antigens from the periodontal lesion can also spread throughout the body.^6^

Data on the prevalence of bacteraemia in dogs and cats undergoing routine dental procedures are limited, although the bacterial species involved have been documented. Blood culture has identified a variety of oral commensals, each associated with differing disease risks. Specifically, in dogs, the incidence of anaerobic bacteraemia was 43%, which was higher than Gram‐positive aerobic bacteraemia (29%) and aerobic Gram‐negative bacteraemia (29%). Despite the lack of veterinary data, to the best of the authors’ knowledge, there is no evidence that dogs and cats experience a greater risk of disease from transient bacteraemia than humans.^7^ It is anticipated that proinflammatory immune mediators produced locally, such as interleukin‐1 (IL‐1), interleukin‐6 (IL‐6), tumour necrosis factor‐alpha (TNF‐α) and prostaglandin E2 (PGE2), enter the systemic circulation and may impact remote organ systems.^5^, ^6^ Therefore, it is essential to consider the overall inflammatory and immune response as a coordinated network rather than individual components working independently.^8^ According to a study conducted in North America, dental disease was the most frequently reported health issue among the feline population.^9^

Stages of PD have been defined by the American Veterinary Dental College (AVDC). These stages range from normal (PD0) to advanced periodontitis (PD4), with each stage defined by the presence or absence of gingival inflammation, attachment loss, furcation involvement and radiological signs of periodontitis. The characteristics of each stage are summarised in Table 1.^1^, ^10^

**TABLE 1:** Stages of periodontal disease (PD) as defined by the American Veterinary Dental College.^10^

Stage	Description	Gingival inflammation	Attachment loss	Furcation involvement	Radiological signs
PD0	Normal	None	None	None	None
PD1	Gingivitis: gingival inflammation without attachment loss	Present	None	None	None
PD2	Early periodontitis: less than 25% attachment loss	Present	<25%	Stage 1 (probe extends < halfway under crown in multirooted teeth)	Radiographic determination of the distance of the alveolar margin from the cementoenamel junction relative to the length of the root
PD3	Moderate periodontitis: 25%‒50% attachment loss	Present	25%‒50%	Stage 2 (probe extends > halfway under crown but not through and through)	Radiographic determination of the distance of the alveolar margin from the cementoenamel junction relative to the length of the root
PD4	Advanced periodontitis: more than 50% attachment loss	Present	>50%	Stage 3 (probe extends under crown through and through from one side to another)	Radiographic determination of the distance of the alveolar margin from the cementoenamel junction relative to the length of the root

Unfortunately, many pet owners and even some veterinarians may not recognise their pets’ clinical signs of PD. Many cats eat normally despite significant infection, inflammation and pain within the oral cavity. Given the prevalence and impact of PD, there is need for a better understanding and management of early‐onset gingivitis and periodontitis in cats.^1^, ^11^ Although regional pain is a known sign of PD in cats, the comprehensive description of systemic effects remains incomplete, even though certain authors assert the presence of substantial effects.^9^ Untreated PD can have severe consequences for both dogs and cats, leading to potential systemic health issues. In dogs, chronic PD is associated with significant complications, such as osteomyelitis with necrotic bone and chronic pulmonary conditions, including chronic obstructive pulmonary disease, fibrosis and emphysema. These findings suggest that PD may contribute to the development of lower airway changes.^12^ Additionally, in humans, a bi‐directional relationship exists between diabetes mellitus and PD, where diabetes increases the risk of PD and effective management of PD can enhance glycaemic control. In geriatric human patients, dental plaque has been linked to pulmonary infections, with periodontitis identified as a risk factor for the development of pneumonia. The bacteraemia associated with PD may lead to distant organ effects and the chronic inflammation from untreated periodontal conditions can result in immune‐mediated changes in various organs.^12^ Diagnosing PD in veterinary cases typically requires clinical examination, including oral examinations, periodontal probing, dental radiography (DTR) and scaling, often under general anaesthesia. While DTR is the most common diagnostic tool, it can underestimate bone loss by up to 1.5 mm in humans due to distortion from inconsistent X‐ray beams. By contrast, computed tomography (CT) may provide more accurate assessments of dental structures.^13^

The systemic effects of PD stem from the dissemination of bacterial metabolic products from periodontopathogens into the bloodstream, eliciting a host response. This response has been associated with elevated systemic inflammatory biomarkers in human patients and dogs.^14^ A study of dogs with PD found a positive correlation between circulating platelet counts and disease severity, as well as a negative correlation with serum creatinine (Cre) levels. However, conventional inflammation indices, such as leukocyte counts and serum C‐reactive protein (CRP) levels, did not show significant changes with treatment.^8^ In humans, the understanding of PD has shifted from the ‘Focal Infection Theory’ to ‘Periodontal Medicine’, which recognises a two‐way relationship between the periodontium and the rest of the body.^6^ In the past five decades, significant advances have been made in understanding the aetiology and pathogenesis of PD and their interactions with the host. This knowledge has illuminated the impact of periodontal conditions on systemic homeostasis, leading to increased understanding that links periodontitis with various systemic diseases. Periodontal medicine, supported by epidemiological evidence and biological plausibility, encompasses the study of how periodontal infections and inflammation can influence extraoral health. Continued progress in this field has focused on enhancing epidemiological methods, statistical power, data analysis techniques and case definitions of periodontitis.^15^ In one study, human individuals diagnosed with chronic periodontitis exhibited a lower count of circulating T cells (CD3‐positive cells) and there was observed reduced in vitro lymphocyte proliferation compared to healthy individuals.^16^ Various systemic indicators of disease associated with periodontitis in humans include elevated levels of acute‐phase proteins and coagulation factors in the bloodstream. This indicates that periodontitis in individuals is associated with systemic inflammation.^6^

Haematological tests are among the most frequently administered laboratory tests in veterinary medicine, and they play a crucial role in diagnosing and managing various health conditions and allow for easy and quick calculation of the relationship between different cells. A simple blood test could be a viable alternative to invasive examinations and costly (and sometimes inaccurate) diagnostic methods such as radiography or CT scan.^9^ Neutrophils are essential components of innate immunity and are the primary circulating phagocytic cells. In the presence of inflammatory stimuli, inflammatory mediators facilitate the migration of immune cells from the circulatory system, allowing them to phagocytose harmful pathogens. Lymphocytes play a pivotal role in orchestrating targeted anti‐inflammatory responses.^17^ Moreover, they are the cells primarily involved in the adaptive immunity response.^18^ Lymphocytes are assumed to decrease during systemic inflammatory response syndrome (SIRS), unlike neutrophils and monocytes, which increase,^19^ and as described, abnormal concentrations of lymphocytes and neutrophils can be seen in the blood of cats with inflammation.^9^ The neutrophil‐to‐lymphocyte ratio (NLR) is a valuable biomarker for evaluating the interplay between innate and adaptive immune responses during inflammatory conditions. Platelets play a crucial role in inflammation by actively coordinating antimicrobial host defense mechanisms and initiating inflammatory responses and tissue repair processes. Thus, the platelet‐to‐lymphocyte ratio (PLR) may serve as a biomarker for the systemic inflammatory response.^17^

The NLR, PLR and monocyte‐to‐lymphocyte ratio (MLR) are cost‐effective and reproducible markers of the systemic inflammatory response. These ratios can be easily calculated from white blood cell (WBC) counts and determined in standard laboratory conditions. As they reflect two immune pathways, these ratios may be less influenced by confounding factors and more predictive in evaluating inflammation than individual immune cell counts.^18^ In feline medicine, NLR has been associated with conditions including mammary carcinoma, hypertrophic cardiomyopathy, SIRS/sepsis, malignant mammary tumours and injection‐site sarcoma size. Combinations of NLR, PLR and MLR have served as survival markers in retroviral infections and indicators in *Cystoisospora*‐associated diarrhoea, with MLR specifically linked to lymphoma outcomes.^19^, ^20^, ^21^, ^22^, ^23^, ^24^, ^25^, ^26^ The NLR/PLR investigated in relation to acute pancreatitis in both dogs and cats, systemic inflammation in canine periodontitis and oropharyngeal tumours, and treatment response in human chronic periodontitis.^17^, ^27^, ^28^

Our study compared NLR, PLR and MLR in cats with PD to those in healthy cats (HC). Our aim was to establish their viability as potential biomarkers for evaluating the systemic inflammatory response caused by PD. This study also seeks to clarify the diagnostic value of haematological indices and systemic inflammatory biomarkers as they relate to gingivitis and advanced stages of PD to evaluate the systemic effects of PD. The development of new diagnostic tests may help to detect active disease, predict disease progression and assess the response to periodontal treatment, ultimately improving the clinical management of periodontal patients.

## MATERIALS AND METHODS

### Animals

The study consisted of 60 client‐owned cats of different breeds, ages and sexes. They were divided into two groups: HCs (*n* = 30) and cats with AVDC stage 3 and 4 PD (PC; *n* = 30). In the HC group, all complete blood count (CBC) parameters were within normal range and all biochemical factors, including liver enzymes, renal parameters, albumin (Alb), globulin, total protein, cholesterol and triglycerides, were normal, in keeping with these cats being considered to be healthy.

The inclusion criteria were cats presented to the Dental Pet Center during a 6‐month period for routine dental check‐ups, dental scaling and PD treatment. Most cats had a history of examination with rapid diagnostic kits for feline leukaemia virus and feline immunodeficiency virus (VDRG) within the past 3 months. If they were not checked, they were tested using rapid diagnostic kits. Before anaesthesia, all cases underwent an oral examination to assess inflammation, calculus, tooth mobility, gingival recession or hyperplasia, halitosis and any abnormalities. In cats that allowed oral examination without anaesthesia, these parameters were assessed during the pre‐anaesthetic evaluation, along with sampling for haematology and serum biochemistry analyses, including a CBC. The biochemical parameters evaluated included alanine transaminase (ALT), aspartate transaminase (AST), blood urea nitrogen, Cre, Alb, total protein, globulin, cholesterol, triglycerides, alkaline phosphatase and fasting blood sugar. The exclusion criteria were cases exhibiting signs of other infectious, inflammatory, immune‐mediated and neoplastic diseases or disorders, including liver or kidney failure, urinary tract disorders, acute upper or lower respiratory problems, anaemia, dermatological diseases, heart disease and gastrointestinal disorders. Moreover, cats that had received any form of medication or undergone surgical procedures within the 3 months before presentation were excluded. The study excluded cats with clinical oral lesions consistent with calicivirus infection or cats exhibiting ocular‒respiratory signs associated with herpesvirus.

### Procedure

Demographic data, including age, neutering status and breed, were recorded. Blood samples were collected from all cases via the jugular vein using a 5 mL syringe (Supa) with a 22‐gauge needle (Supa) measuring 32 mm in length. Approximately 3‒4 mL of blood was collected; 1 mL was transferred into a tube containing EDTA for CBC analysis (Farzaneh Arman), and the remaining 2‒3 mL was transferred into a Gel and Clot Activator tube (Vacutest Kima SRL) for biochemical parameter assessment (Mindray BS‐200E). After evaluating the blood parameters and confirming the animals' adequate health status for anaesthesia, all cases were anaesthetised and underwent radiography and oral examination. An intravenous line was established for all cases. Anaesthesia was induced using 1 mg/mL medetomidine (Zoetis) (0.12 µg/kg), 10 mg/2 mL diazepam (Caspian Tamin) (0.2 mg/kg) and 10% ketamine (Bremer) (7 mg/kg). Maintenance of anaesthesia following intubation was achieved with inhalation anaesthesia using isoflurane gas (2% end‐tidal concentration) (Piramal Critical Care) in 100% oxygen.

### Measured parameters

The evaluated indices in both study groups included gingivitis, probing depth, furcation exposure and tooth mobility. Therefore, when furcation involvement, probing depth and tooth mobility were observed, radiographs were used to confirm alveolar bone loss greater than 25%‒50% of the root heights. Cats entered the study after clinical and radiographic evaluations demonstrated they were affected with advanced periodontitis, which is consistent with AVDC stage 3 or 4 PD. The total WBC, neutrophil, lymphocyte, monocyte and eosinophil counts in both groups were measured (Nihon Kohden Celltac MEK‐6450K) and the NLP, PLR and MLR were then calculated.

### Statistical analysis

Statistical analyses were conducted using the Statistical Package for the Social Sciences software (version 27; IBM Corp). The Shapiro‒Wilk test was used to assess the data for normality. Because the data were normally distributed, an independent sample *T*‐test was used to compare all data between HC and cats with stages 3 and 4 PD. The demographic data were evaluated using the chi‐square test. A *p‐*value of less than 0.05 was considered statistically significant.

## RESULTS

### Demographic data

Table 2 shows the demographic data from the studied cats, including age and neutering status. There were no significant differences in demographic data between the groups. In the HC group, 40% were Persian, 3.33% were British and 56.66% were mixed‐breed cats. In the PC group, 43.3% were Persian, 3.33% were British, 3.33% were Scottish and 50% were mixed‐breed cats.

**TABLE 2:** Demographic data of a group of healthy cats (HC, *n* = 30) and cats with periodontal disease (PC, *n* = 30), compared to observe difference in haematological variables.

Group	Age of cats (years, mean and standard deviation)	No. (%) of spayed female cats	No. (%) of castrated male cats	No. (%) of sexually intact female cats	No. (%) of sexually intact male cats
HC (*n* = 30)	5.39 ± 4.03	6 (20)	12 (40)	5 (16.66)	7 (23.33)
PC (*n* = 30)	4.80 ± 3.34	4 (13.3)	11 (36.66)	5 (16.66)	10 (33.33)
*p*‐Value	0.48	0.48	1.00	1.00	0.39

### Haematological analysis

Table 3 summarises data regarding the haematological analysis. For NLR, PLR and MLR, there was no statistically significant difference between the two groups HC and PC (*p *> 0.05).

**TABLE 3:** Mean ± standard deviation of haematological parameters measured in two healthy cats (HC, *n* = 30) and cats with periodontal disease (PC, *n* = 30).

Haematological parameter	HC group	PC group	*p*‐Value
Total white blood cell count (×10^3^/µL)	8.24 ± 5.27	10.24 ± 8.27	0.17
Platelets (×10^3^/µL)	308.58 ± 169.88	298.70 ± 154.85	0.79
(×10^3^/µL) Neutrophil	4.90 ± 3.89	6.66 ± 6.80	0.14
Lymphocyte (×10^3^/µL)	2.46 ± 1.72	2.48 ± 1.58	0.93
Eosinophil (×10^3^/µL)	0.59 ± 0.58	0.55 ± 0.61	0.97
Monocyte (×10^3^/µL)	0.22 ± 0.23	0.27 ± 0.26	0.40
Neutrophil/lymphocyte	2.69 ± 2.70	3.71 ± 5.16	0.25
Platelet/lymphocyte	179.33 ± 151.78	178.69 ± 152.05	0.86
Monocyte/lymphocyte	0.097 ± 0.069	0.152 ± 0.17	0.10

## DISCUSSION

The present study did not find any significant difference between haematological biomarkers in HC and PC cats, indicating that PD did not notably impact on these blood parameters in these cases.

A previous study reported a direct relationship between factors such as ALT and immunoglobulin G (IgG) and the severity of PD in cats, as well as an inverse relationship with haemoglobin, haematocrit and AST.^9^ They assessed the severity of PD by scoring dental calculus, gingivitis and periodontitis, using an adapted scoring system adapted as previously reported.^29^ The mean ± SD scores for disease severity in both groups combined were gingivitis, 2.2 ± 0.4 (out of 4); calculus, 1.5 ± 0.26 (out of 3); mean alveolar bone resorption, 14.9 ± 3.9 mm; and average alveolar bone resorption, 1.5 ± 0.35 mm. In their study, cats with periodontitis were divided into two groups: one was left untreated and the other was treated. After the treatment period, IgG, total globulin and AST significantly decreased while cholesterol increased. However, contrary to what was observed in human studies, the number of other leukocytes did not show a significant difference between pre‐ and post‐treatment. While the authors noted the lack of a comparative healthy control group in their study, their comparison of treatment and control groups found no significant association between total neutrophil, monocyte and lymphocyte counts, and dental treatment.^9^, ^29^ This absence of a significant difference in total WBC counts is consistent with our results.

Periodontal disease in cats can manifest as either aggressive (the rare form of inflammation characterised by a rapid evolution, mainly observed in young adults) or chronic (widespread slow‐progressing forms of periodontal inflammation).^30^ The chronic inflammatory condition may or may not be associated with an increase in the number of WBCs. Previous studies have reported that humans with PD exhibit higher WBC counts than healthy individuals, whereas the same correlation was not observed in dogs.^8^, ^17^ Additionally, NLR, MLR and PLR have been proposed as diagnostic and prognostic markers for neoplastic and inflammatory diseases in dogs and cats.^31^ Even so, there were no significant differences between our study groups in relation to the selected haematological markers.

In cats, NLR has been reported to be increased in certain diseases, such as mammary tumours,^20^, ^23^ hypertrophic cardiomyopathy,^21^ and SIRS and sepsis.^32^ One study reported that PD had no impact on the NLR in dogs. This inflammatory and immune‐mediated disease in dogs may be associated with a well‐balanced innate and adaptive immune response displayed by neutrophils and lymphocytes. However, in dogs with oral tumours, the NLR was significantly higher. This suggests that the NLR may increase in response to the inflammatory nature of neoplastic conditions rather than inflammatory head and neck conditions such as PD.^17^ However, our study found that periodontitis did not significantly change the NLR and other haematological biomarkers.

There are few studies on MLR in cats, including one that reported a decrease in LMR in cats with infectious, neoplastic and chronic kidney disease, accompanied by an increased NLR.^31^ There is a study of cats with diarrhoea and infected with *Cystoisospora* spp., where MLR and NLR were elevated. The authors of that study stated that they were the first to measure MLR in cats.^19^ In contrast, the increase in monocyte levels was negligible in our study and no statistically significant difference in the MLR was observed between the two groups.

It was expected that some indices, such as the number of platelets in the PD group, would be lower than in the HC group, and a difference would be observed since a significant portion of animals in the PD group had a history of bleeding from the gums. However, an insignificant increase in platelets could be expected as reported in a study of young human patients with stage 3 grade C periodontitis^33^; traditionally, platelets have been recognised primarily for their role in haemostasis. Even so, they also significantly contribute to inflammatory processes, with platelet counts often rising during chronic inflammation.^34^ This increase in platelet levels is notably correlated with gingivitis and attachment loss for two main reasons.^8^ First, PD causes the degradation of the gingival sulcular epithelium and associated vascular endothelium, leading to frequent haemorrhage in the affected tissue. This persistent injury activates the coagulation cascade, particularly in advanced cases of PD. Second, the close relationship between platelets and the inflammatory response, as well as their involvement in wound healing, may facilitate the repair of damaged sulcular epithelium, thereby stimulating megakaryocyte activity. The observed lack of response post‐treatment could be attributed to prolonged stimulation of the bone marrow, which may take over 30 days to normalise.^8^ After all, the reduction in platelet count in our study was slightly prominent, although it was not statistically significant—possibly due to the bleeding.

A study reported that cats with PD exhibited elevated numbers of mast cells in their gingiva. This suggests that the immune response in feline PD may involve mast cell activation, which could contribute to the chronic inflammatory process and tissue destruction observed in the condition. Further exploration of the role of mast cells in feline PD could offer valuable insights into potential therapeutic targets for managing the inflammatory response and preventing tissue damage.^35^

This study had several limitations that need to be recognised. First, we did not conduct a retrospective power analysis to determine if the sample size was adequate for identifying statistically significant differences. Future studies that include power analysis calculations before data collection would help guarantee sufficient sample sizes to detect possible associations. Second, this study concentrated on certain haematological parameters, including NLR, PLR and MLR. Other inflammatory indicators, such as CRP and proinflammatory cytokines (such as IL‐6 and TNF‐α), were not assessed. Incorporating these biomarkers in future studies may offer a more thorough evaluation of systemic inflammation linked to PD.

It can be concluded that there was no significant difference between the measured haematological biomarkers in HC and PD cats, including NLR, MLR and PLR. Thus, other concurrent diseases or inflammatory causes should be considered if significant changes are found.

## AUTHOR CONTRIBUTIONS

*Investigation, methodology and data curation*: Hossein Ghorbani. *Conceptualisation, methodology, data curation, investigation, and review and editing*: Azin Tavakoli. *Data curation, investigation and methodology*: Mohammad Heidarpour. *Validation, writing and editing*: Negin Rahimdoust Mozhdehi. *Methodology, investigation, supervision, validation, visualisation, and review and editing*: Hossein Kazemi Mehrjerdi.

## CONFLICTS OF INTEREST

The authors declare they have no conflicts of interest.

## FUNDING INFORMATION

The authors received no specific funding for this Work.

## ETHICS STATEMENT

The authors confirm that the journal's ethical policies, as noted on the journal's author guidelines page, have been adhered to. The research was approved by the Bioethics Committee of Ferdowsi University of Mashhad (IR.UM.REC.1400.364). Information was given to the owners and written consent was obtained.

## Data Availability

The data that support the findings of this study are available from the corresponding author upon reasonable request.
